# Partial Transfer Learning Method Based on Inter-Class Feature Transfer for Rolling Bearing Fault Diagnosis

**DOI:** 10.3390/s24165165

**Published:** 2024-08-10

**Authors:** Hongbo Que, Xuyan Liu, Siqin Jin, Yaoyan Huo, Chengpan Wu, Chuancang Ding, Zhongkui Zhu

**Affiliations:** 1 School of Mechanical, Electronic and Control Engineering, Beijing Jiaotong University, Beijing 100044, China; quehbff@126.com; 2 CRRC Qishuyan Locomotive and Rolling Stock Technology Research Institute Co., Ltd., Changzhou 213003, China; jinsiq@163.com (S.J.); huoyaoyan@126.com (Y.H.); wuchengpan@126.com (C.W.); 3 School of Rail Transportation, Soochow University, Suzhou 215137, China; lxy_work2021@163.com (X.L.); zhuzhongkui@suda.edu.cn (Z.Z.)

**Keywords:** fault diagnosis, partial transfer learning, label imbalance, domain adaptation, deep transfer learning

## Abstract

Rolling bearing fault diagnosis methods based on transfer learning always assume that the sample classes in the target domain are consistent with those in the source domain during the training phase. However, it is difficult to collect all fault classes in the early stage of mechanical application. The more likely situation is that the training data in the target domain only contain a subset of the entire health state, which will lead to the problem of label imbalance compared with the source domain. The outlier classes in the source domain that do not have corresponding target domain samples for feature alignment will interfere with the feature transfer of other classes. To address this specific challenge, this study introduces an innovative inter-class feature transfer fault diagnosis approach. By leveraging label information, the method distinctively computes the distribution discrepancies among shared classes, thereby circumventing the deleterious influence of outlier classes on the transfer procedure. Empirical evaluations on two rolling bearing datasets, encompassing multiple partial transfer tasks, substantiate that the proposed method surpasses other approaches, offering a novel and efficacious solution for the realm of intelligent bearing fault diagnosis.

## 1. Introduction

Rolling bearings are an indispensable component of modern machinery, playing a crucial role in mechanical transmission systems by reducing friction, supporting rotating parts, and ensuring precise operation of equipment. The efficient functioning of rolling bearings is vital to the performance of the entire mechanical system, with their health status directly affecting the stability, reliability, and overall production efficiency of the equipment. When rolling bearings fail, it can lead not only to shutdowns or slowdowns of the production line, impacting the production schedule and economic benefits of the enterprise, but also, due to knock-on effects of the failure, cause damage to other components. In some cases, it may even trigger serious safety accidents. Therefore, ensuring the healthy operation of rolling bearings and timely detection of potential anomalies and faults is crucial for avoiding these risks.

To effectively monitor and diagnose the health status of rolling bearings, researchers and enterprises have invested substantial resources in developing intelligent monitoring technologies. With the rapid development of artificial intelligence technologies, especially breakthroughs in the field of machine learning, new possibilities for intelligent fault diagnosis of rolling bearings have been provided. Machine learning methods identify patterns and regularities in data by training algorithmic models, capable of learning fault characteristics from a vast amount of historical data and predicting future performance changes [1,2,3,4,5]. Widely used machine learning methods include decision trees [6], random forests [7], support vector machines [8], artificial neural networks [9], and deep learning, etc. In particular, deep learning can automatically extract complex features and process high-dimensional and unstructured data, such as raw vibration signals, by constructing deep neural networks. This technology eliminates the need for manual feature design, enabling it to learn useful information directly from raw data, greatly improving the accuracy and efficiency of fault detection. In addition, deep learning models have demonstrated exceptional capabilities in classification problems, accurately distinguishing between normal states and various types of fault conditions, aiding maintenance personnel in rapidly locating faults, and implementing appropriate repair measures. Algorithms frequently used in the field of deep learning include convolutional neural networks (CNN), deep belief networks [10], deep autoencoders [11], and long short-term memory networks [12], among others. Li et al. [13] researched a rolling bearing fault diagnosis algorithm using derivatives and enhanced discrete analytical wavelets. They obtained a morphological spectrum of the fault signal through multi-scale morphological opening operations. The morphological spectrum entropy was calculated from the morphological spectrum curve to describe the morphological characteristics of different signals of rolling bearings. The signal’s morphological feature decomposition was carried out by approximate analytical complex wavelet transformation, wavelet packet space rearrangement, and cross-combination of wavelet packet spaces. Chen et al. [14] analyzed the lower boundary of the two-dimensional data input size by considering the relationship between the characteristic vibration frequency and the short-time Fourier transform window size, guiding the determination of the minimum input size. Then, they designed a universal adaptive CNN structure for different datasets. Hu et al. [15] proposed a method combining multi-scale autoencoders with generative adversarial networks to extract deep sensitive features of signals, which were then combined with a classifier for fault diagnosis. Gu et al. [16] proposed a multi-sensor fault diagnosis model based on long short-term memory and discrete wavelet transform. Initially, detailed fault information at different frequencies and time scales was obtained using wavelet transformation; subsequently, a long short-term memory network characterized the long-term dependencies hidden in the fault information time series. This method fully leveraged the advantages of feature extraction based on expert experience and deep network learning, enabling the discovery of complex patterns from a vast amount of data. Therefore, under the context of Industry 4.0, rolling bearing health monitoring and fault diagnosis technologies based on deep learning has become an essential tool for enhancing the level of equipment intelligence, ensuring production safety, and increasing production efficiency.

Despite this, applying these methods to real-world scenarios still encounters significant challenges. Ideally, the training and test sets should follow the same data distribution; however, in actual work environments, variability and noise often cause distribution discrepancies between datasets, leading to domain shift [17], severely compromising diagnostic accuracy. To address this issue, deep transfer learning (DTL) [18], a strategy that allows knowledge learned from other domains to be applied to new diagnostic tasks, is garnering increasing attention in the field of bearing fault diagnosis and has become an effective tool for intelligent diagnosis of bearings under variable conditions. Currently, researchers have developed various DTL models [19], which can roughly be categorized into model-based DTL [20], instance-based DTL [21], mapping-based DTL [22], and adversarial-based DTL [23]. Model-based DTL assumes that a portion of the parameters from the model trained using source domain data can be fine-tuned and applied to classification tasks in the target domain. Cao et al. [24] proposed a transfer learning architecture composed of two parts; the first part consists of a pre-trained deep neural network capable of automatically extracting features from inputs, and the second part is a fully connected network that classifies the extracted features using gear fault experimental data. Instance-based DTL involves selecting specific samples from the source domain using a particular weight adjustment strategy, assigning appropriate weights to them, and then integrating these weighted samples into the target domain dataset for model training in the target domain. Chen et al. [25] introduced auxiliary labeled data and target labeled data to form a combined training set and applied heterogeneous distribution weighted random sampling to reorganize the training set, obtaining an optimized combined training set that is “approximately the same distribution” as the test set, achieving accurate classification of bearing faults under different working conditions. Mapping-based DTL projects features from the source and target domains into a shared space to identify and confirm the common latent attributes between the two domains, which then act as a bridge linking different domains. Maximum mean discrepancy (MMD) is a commonly used mapping method. Yang et al. [26] proposed a polynomial kernel-based MMD distance measure to overcome the shortcomings of the widely applied MMD. Adversarial-based DTL encourages the model to recognize transferable features applicable to both the source and target domains through the principle of adversarial learning. Adversarial-based DTL encourages the model to recognize transferable features applicable to both the source and target domains through the principle of adversarial learning. Han et al. [27] proposed a new deep adversarial convolutional neural network. Adding a discriminative classifier forces the feature extractor to extract common features shared by both domains, enhancing the model’s generalization ability.

However, despite the notable advantages of deep transfer learning in the field of intelligent fault diagnosis, its application still faces significant constraints. These methods often rely on a core assumption: that the source and target data domains should share the same label set. This is frequently difficult to achieve in practice, especially during the initial operation phase of new equipment or under new working conditions, where there is a lack of sufficient representative monitoring data to depict the various possible health states, making it challenging to construct a complete target label space. Consequently, when the label spaces of the source and target domains are not consistent, models based on the shared label assumption encounter difficulties in adapting to the actual working environment.

To address this practical challenge, a partial transfer learning method for bearing fault diagnosis based on inter-class feature transfer is proposed in this paper, which uses label information from the target domain to perform partial domain adaptation. This enables the model to comprehensively cover and monitor all health states under new working conditions, adapting more effectively to the various needs and challenges of real-world environments.

The organizational structure of this paper is as follows. Section 2 describes the theoretical methods of JS divergence and AM-Softmax. The proposed approach is described in detail in Section 3. In Section 4, two datasets are used to verify the effectiveness of the proposed method. Finally, the conclusion is summarized in Section 5.

## 2. Preliminaries

### 2.1. Jensen–Shannon Divergence

The Jensen–Shannon divergence (JS) is a method for measuring the similarity between two probability distributions and is based on the Kullback–Leibler divergence (KL). However, unlike KL, it is symmetric and always bounded. Given two probability distributions P and Q, the JS is defined as the average of the KL between each distribution and their average $M=\frac{1}{2}\left(P+Q\right)$:

(1) $$JS\left(P\|Q\right)=\frac{1}{2}D_{KL}\left(P\|M\right)+\frac{1}{2}D_{KL}\left(Q\|M\right)\;$$

where $D_{KL}$ represents the KL divergence, and the formula for $D_{KL}\left(P\|Q\right)$ is as follows:

(2) $$D_{KL}\left(P\|Q\right)=\sum_{i}P\left(i\right)\log\left(\frac{P(i)}{Q(i)}\right)\;$$

Therefore, the specific formula for the JS is as follows:

(3) $$JS\left(P\|Q\right)=\frac{1}{2}\sum_{i}\left[P\left(i\right)\log\left(\frac{P(i)}{M(i)}\right)+Q\left(i\right)\log\left(\frac{Q(i)}{M(i)}\right)\right]\;$$

In summary, the JS was introduced to address the limitations of the KL. It holds significant value in measuring the differences between probability distributions, particularly within the field of machine learning. As a loss function, it aids in optimizing models to better match the target distribution.

### 2.2. Additive Margin Softmax

The traditional Softmax loss function is widely used in classification problems; it is essentially a combination of the Softmax function and the cross-entropy loss function. The expression for this loss function can be written as follows:

(4) $$\begin{array}{c} L_{S}=-\frac{1}{n}\sum_{i=1}^{n}\log\frac{e^{W_{y_{i}}^{T}f_{i}}}{\sum_{j=1}^{c}e^{W_{j}^{T}f_{i}}}\\ =-\frac{1}{n}\sum_{i=1}^{n}\log\frac{e^{∥W_{y_{i}}∥∥f_{i}∥\cos\left(\theta_{y_{i}}\right)}}{\sum_{j=1}^{c}e^{∥W_{y_{i}}∥∥f_{i}∥\cos\left(\theta_{y_{i}}\right)}} \end{array}$$

where $f_{i}$ denotes the input of the *i*-th sample in the last fully connected layer, $W_{j}$ represents the *j*-th column of the last fully connected layer, $e^{W_{y_{i}}^{T}f_{i}}$ is the predicted score for the *i*-th sample being the correct class, and $\sum_{j=1}^{c}e^{W_{y_{i}}^{T}f_{i}}$ is the predicted score for all classes. The equation below is an expansion of the dot product formula from the above. A key advantage of the Softmax loss function is its ability to effectively differentiate between different classes. However, it performs poorly when dealing with distances between samples within the same class, sometimes even leading to intra-class distances that exceed inter-class distances. This can significantly affect the alignment of marginal distributions during feature transfer.

**Figure 1:**
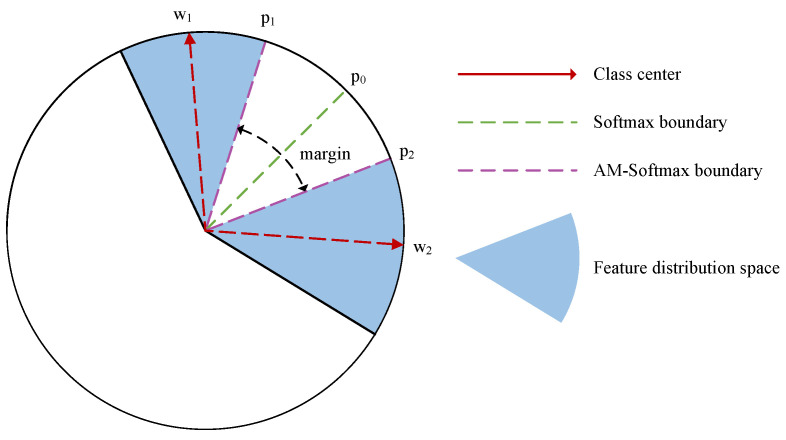
The difference between AM-Softmax and traditional Softmax lies in the introduction of a margin by AM-Softmax, which enhances the separability between classes and makes the samples within the same class more compact.

The Additive margin Softmax (AM-Softmax) [28] introduces improvements upon the standard Softmax by normalizing both the weights and input features, incorporating two hyperparameters. The resulting loss function expression is as follows:

(5) $$L_{\mathrm{AMS}}=-\frac{1}{n}\sum_{i=1}^{n}\log\frac{e^{s\cdot (\cos\theta_{y_{i}}-m)}}{e^{s\cdot (\cos\theta_{y_{i}}-m)}+\sum_{j=1,j\neq y_{i}}^{c}e^{s\cdot \cos\theta_{y_{i}}}}$$

where *s* is the scaling factor, and *m* is the scale factor that determines the degree of inter-class clustering. Figure 1 compares the impact of AM-Softmax with standard Softmax on data distribution in feature space. With traditional Softmax, the decision boundary is at $p_{0}$, whereas for AM-Softmax, the boundaries between class 1 and class 2 are at $p_{1}$ and $p_{2}$, respectively, with a margin area in between ensuring discrimination between the two classes.

Using the AM-Softmax loss can significantly enhance the aggregation of fault signals in feature space, thereby increasing the distributional differences between intra-class samples and inter-class samples. This improvement helps facilitate feature transfer for the same class within different distributions.

## 3. Proposed Method

### 3.1. Problem Formulation

In the field of transfer learning, one of the core challenges is dealing with the differences in marginal distributions between the source domain and the target domain. The goal is to enable a model trained using a large amount of labeled data in the source domain to generalize more effectively in the target domain. Traditional transfer learning generally assumes that the source domain and the target domain share the same label space, meaning that the classes contained in the datasets are consistent, allowing for corresponding matching between each class in the source domain and the corresponding class in the target domain during training. However, in scenarios of partial transfer learning (PTL), unique outlier classes in the source domain may adversely affect the distribution matching between shared classes.

**Figure 2:**
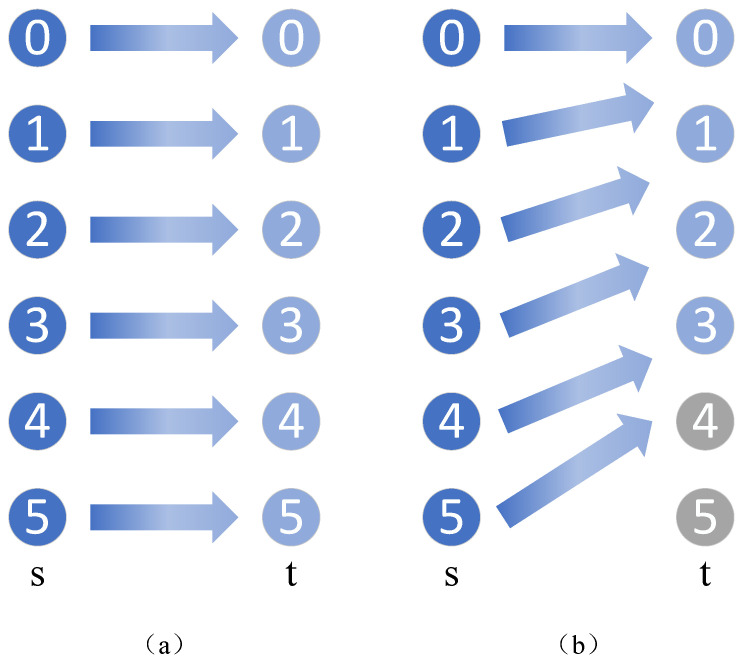
Negative impact of the outlier class. (**a**) Traditional transfer learning. (**b**) Partial transfer learning.

Figure 2 provides an intuitive example. In Figure 2a, a typical scenario is shown on how to align marginal distributions in traditional transfer learning, where each source domain class can find a corresponding target domain class. However, in the situation shown in Figure 2b, “Class 4” and “Class 5” in the source domain are outlier classes that, if left unchecked, may adversely affect the transfer learning process of other classes, which is undesirable.

Under the PTL framework, it is often assumed that the source domain dataset is complete, or even over-complete, meaning that it contains a wide range of classes, a large number of samples, and accurate labels, forming an idealized dataset. In contrast, the label space of the target domain is merely a subset of the source domain’s label space. Some research in PTL focuses on enhancing the model’s classification ability for certain specific classes in new scenarios, which usually occurs when source domain information is too complex leading to negative transfer effects, and the target domain lacks labels making it impossible to manually select appropriate source domain data. Other studies focus on improving the model’s classification ability across all classes, aiming to use an informationally complete source domain and an informationally incomplete target domain to enable the model to accurately classify all classes on the target domain distribution. The problem background focused on in this paper is the latter description.

This research is conducted under the following assumptions:

1. The health status labels in the source domain are sufficient to cover all possible states in the target domain;
2. Each class within the source domain has an ample amount of labeled samples;
3. Each class within the target domain has an ample amount of labeled samples;
4. There are differences between the source and target domains in terms of data distribution and label space.

Given two datasets $D_{S}={\left\{\left(x_{i}^{s},y_{i}^{s}\right)\right\}}_{i=1}^{n_{s}}$ and $D_{T}={\left\{\left(x_{i}^{t},y_{i}^{t}\right)\right\}}_{i=1}^{n_{t}}$, representing the source domain and target domain datasets, respectively, $y_{s}$ and $y_{t}$ are the labels of the source domain and target domain, respectively, while $n_{s}$ and $n_{t}$ represent the sample sizes of the source domain and target domain. $P_{s}\left(x,y\right)$ and $P_{t}\left(x,y\right)$ are the data distributions of the source domain and target domain, $P_{s}\left(x,y\right)\neq P_{t}\left(x,y\right)$. $C_{s}$ and $C_{t}$ are the sets of classes for the source domain and target domain, $C_{t}\subseteq C_{s}$.

### 3.2. Inter-Class Feature Transfer Module

During the training process, since the sample labels of the target domain are obtainable, it becomes very clear which classes are outliers. The inter-class feature transfer module (ICFT) fully utilizes the label information from both domains to mitigate the potential negative effects of outlier classes from the source domain during the transfer process. ICFT utilizes the Jensen–Shannon divergence to describe the degree of similarity between pairs of classes from the two domains. The formula for the ICFT loss is as follows:

(6) $$L_{ic}=\frac{1}{\sum_{i=1}^{k}n_{i}}\sum_{i=1}^{k}JS(X_{i}^{s}\|X_{i}^{t})$$

**Figure 3:**
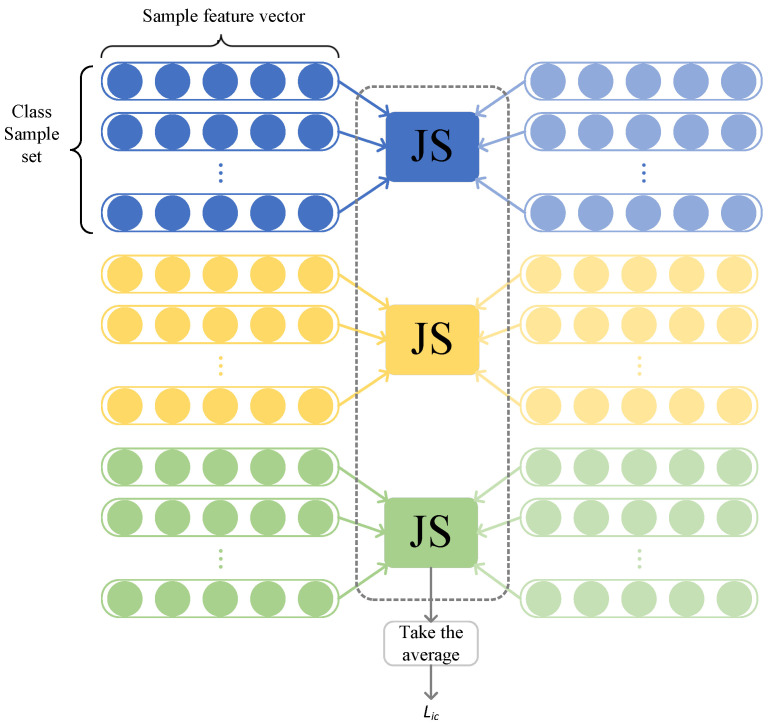
Inter-class feature transfer module logic.

Here, *k* refers to the number of classes shared between the source and target domains. $n_{i}$ represents the total number of samples for a specific class (i.e., class *i*). $X_{i}^{s}$ and $X_{i}^{t}$ represent the sets of samples from class *i* in the source and target domains, respectively. When calculating the ICFT loss, only the Jensen–Shannon divergence between the same classes in both domains is considered, ignoring the relationships between different classes. The main purpose of this approach is to prevent confusion between features of different classes during the feature transfer process. In practice, to capture the similarity between two classes more accurately, the sample pairs from both domains are randomly shuffled each time when calculating the ICFT loss. The logical structure of ICFT is as shown in Figure 3.

### 3.3. General Framework

**Figure 4:**
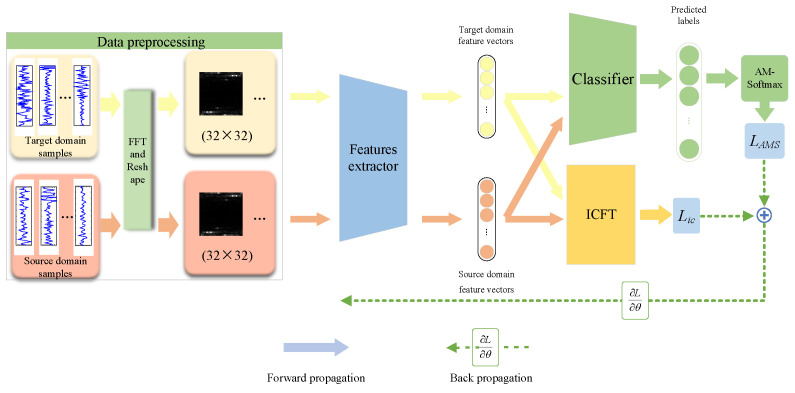
The framework of the proposed method.

The overall framework of the method is shown in Figure 4.

The original rolling bearing vibration signals are first processed through a fast Fourier transform and converted into two-dimensional grayscale images, which serve as the input data for the model. The feature extractor within the model utilizes a CNN network to extract features. Subsequently, the feature extractor derives high-dimensional feature vectors from samples of both the source and target domains, which are then transmitted to the inter-class feature transfer module and the classifier, respectively. When addressing the image classification problem of bearing health conditions, it is important to recognize that the similarity between different health states is generally higher than in other types of image classification tasks. This implies that in the feature space, the distributions of different health state classes are closer to each other. Therefore, to effectively distinguish these states, it is crucial to increase the margin between classes and enhance the compactness within the same class. The loss $L_{AMS}$ is calculated using AM-Softmax. Compared to the traditional cross-entropy loss, AM-Softmax can significantly enhance the clustering effect between classes. The designed inter-class feature transfer module utilizes label information to select samples of shared classes from both the source and target domains, and independently calculates the Jensen–Shannon divergence between the two domain samples for each pair of classes, thereby obtaining the loss $L_{ic}$. This process eliminates the interference of outlier classes in the source domain, helping the model to more accurately align the relevant class features between the source and target domains.

### 3.4. Loss Function and Optimization

Combining Equations (5) and (6), the overall loss *L* can be derived.

(7) $$L_{ic}=\frac{1}{\sum_{i=1}^{k}n_{i}}\sum_{i=1}^{k}JS(X_{i}^{s}\|X_{i}^{t})$$

To minimize the loss function (7) and update the parameters in the network model, a backpropagation algorithm combined with the Adam optimizer is employed here. Let $\theta_{f}$ and $\theta_{c}$ represent the parameters of the feature extractor and classifier, respectively; the parameter update process is described as follows:

(8) $$\theta_{f}\leftarrow \theta_{f}-\varepsilon \left(\frac{\partial L_{ic}}{\partial \theta_{f}}+\frac{\partial L_{AMS}}{\partial \theta_{f}}\right)$$

(9) $$\theta_{c}\leftarrow \theta_{c}-\varepsilon \frac{\partial L_{AMS}}{\partial \theta_{f}}$$

where θ represents the learning rate, which is set to a default value of $10^{-3}$.

## 4. Experiment Results

### 4.1. Dataset Description

**Figure 5:**
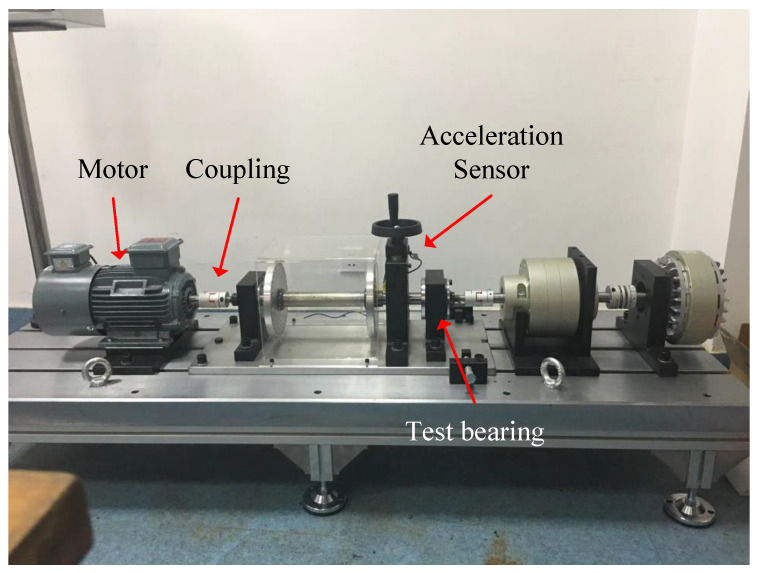
Rolling bearing test bench.

Experiments were conducted on two datasets to verify the proposed method’s effectiveness. Dataset I: The dataset from reference [29] is used. Collected from the bearing test bench shown in Figure 5.

**Figure 6:**
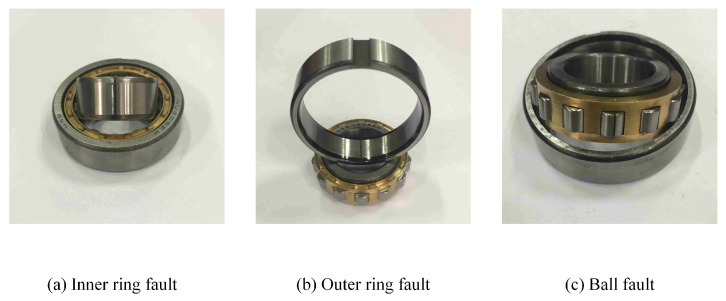
Three bearing failure types.

The bearing model used in the test rig is N205EU. Cracks were cut on the bearing rollers, inner rings, and outer rings using wire-cutting technology to simulate partial failures of the bearing, as shown in Figure 6. The experimental design included 10 different sample classes, normal condition (H), inner ring fault (IF), ball fault (BF), and outer ring fault (OF), with each fault type further divided into three levels based on crack width: 0.2 mm, 0.4 mm, and 0.6 mm. The rotation speed was set at 2000 revolutions per minute, with loads set at 0 N, 20 N, 40 N, and 60 N, respectively.

**Figure 7:**
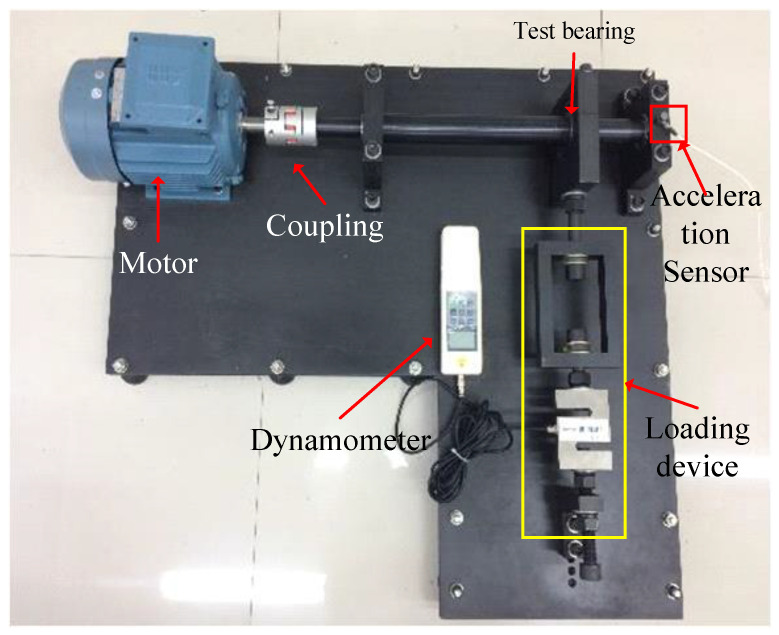
Self-made rolling bearing test bench.

Dataset II: Collected from the rolling bearing test bench made in the laboratory, as shown in Figure 7.

**Figure 8:**
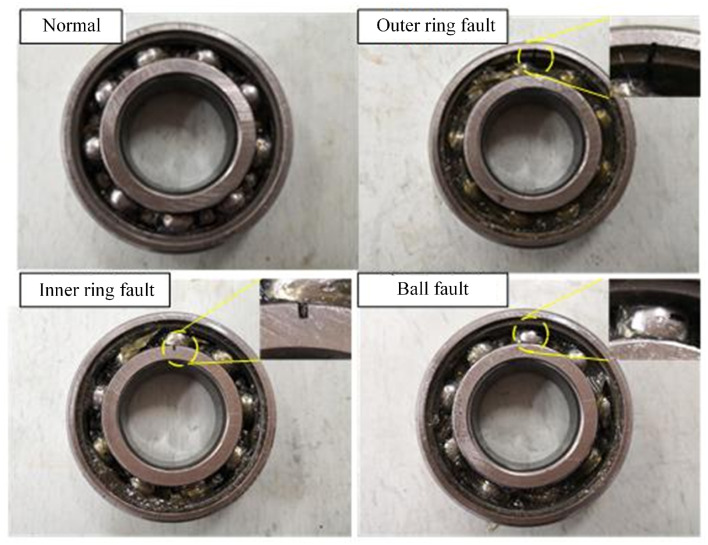
Four bearing health states.

The test bench mainly consists of a drive motor, coupling, bearings, load cell, and accelerometer. The bearing model used in the experiment is 6205-2RS (Svenska Kullager-Fabriken Co. Ltd., Gothenburg, Sweden). Different types of fault data were obtained by replacing bearings with various faults. The load size can be changed by adjusting the nut and measured by the load cell. The motor controls the rotation speed, which can be adjusted within 800 to 1200 revolutions per minute. To mimic partial failure conditions of the bearing, cracks were cut on the rollers, inner rings, and outer rings of the bearing using wire cutting technology, as shown in Figure 8, with crack widths of 0.2 mm, 0.4 mm, and 0.6 mm. In collecting experimental data, the rotation speed was set at 896.1 revolutions per minute, and the sampling frequency was 10.6 kHz. By adjusting the nut, loads were set at 1 kN, 2 kN, and 3 kN, respectively. The experimental design included 10 different sample classes: normal condition (H), inner ring fault (IF), ball fault (BF), and outer ring fault (OF), with each fault type further divided into three levels based on crack width: 0.2 mm, 0.4 mm, and 0.6 mm.

The detailed information on the fault classes and labels of the two datasets is shown in Table 1.

In this study, different domains are divided according to different load conditions. Dataset I covers six load domains, with 12 transfer learning tasks conducted between them for experimental research. Dataset II covers three load domains, with six transfer learning tasks conducted between them for experimental research. In the experiments, the source domain dataset contains all 10 sample classes, with 200 training samples provided for each class. However, the target domain dataset is limited to accessing only five classes of samples, specifically classes 0, 3, 7, and 9, with each class also having 200 training samples. During the testing phase, the remaining 200 samples from all 10 classes in the target domain are used for test evaluation. For network training, this study chose the Adam algorithm to update the network parameters. To ensure a fair comparison, all comparative experiments utilized the same CNN network as the backbone network and set a unified random seed to ensure consistency in the initialization parameters of the network, thereby reducing random errors in the experimental results. Each transfer task was independently repeated 10 times, and the final results were taken as the average of these 10 experiments to ensure the stability and reliability of the results. Detailed information on all transfer tasks on both datasets has been listed in Table 2.

### 4.2. Results and Analysis

To verify the effectiveness and superiority of the designed methods, several classic methods were selected for comparison, as shown in Table 3. M1 corresponds to a conventional supervised learning model that uses only source domain data and applies the standard cross-entropy loss function. M2 includes target domain samples in the training set while continuing to use the cross-entropy loss. M3, building on M2, adds domain adaptation loss by incorporating MMD to match the feature distributions of the two domains. M4 is a traditional adversarial-based transfer learning method. M5 and M6 are classical methods for partial domain adaptation. M7 is an advanced method for domain adaptation, and M8 eliminates the target domain labels based on M7. A1 replaces the loss function with AM-Softmax based on M2. ICFT integrates all the strategies proposed in this paper. Table 4 and Table 5 compare the performance of different methods on two datasets. It can be seen that the proposed method has achieved leading results in most transfer tasks and has reached the highest average performance of 84.55% and 96.23% across all tasks on both datasets. The A1 algorithm, which only adopts the AM-Softmax loss function without using the inter-class feature transfer module, has an average performance of 83.00% and 94.70%, respectively, thereby confirming the importance of the inter-class feature transfer module in enhancing the overall model performance. The M2 method, which can access target domain data, outperforms the M1 method, which can only use source domain data, by 11.25% and 5.1% in performance, highlighting the necessity of addressing the domain shift problem. However, compared to M2 without feature transfer, M3 and M4 do not show significant performance improvements or even decreases. Especially in Dataset I, M4’s performance decreases by 20.42% compared to M2. This may be due to the adverse effects of outlier class samples from the source domain during the transfer process. M8 is a newer and more advanced domain adaptation method than M3 and M4, yet it lags behind M3 by 5.18% in performance in Dataset I. The training process of M7 does not involve target domain labels and also suffers a significant performance loss compared to M8. Notably, although M5 and M6 are deep transfer learning methods optimized for label imbalance problems, they perform the worst among all methods. The reason for this is that the partial transfer issues focused on by these two methods differ from those defined in this paper. They focused solely on the model’s ability to classify within the target domain’s label space, overlooking its capability to classify outlier classes in the source domain. The strategy of M5 and M6 is to identify outlier classes during training and assign them lower weights to reduce their impact on the transfer process. However, when the input data include labels, the model quickly identifies outlier class samples and assigns them extremely low weights, causing the model to fail to learn the features of these classes. The information about outlier classes learned from the source domain in the early training stage suffers from catastrophic forgetting and is quickly overridden by information related to other shared classes. This contradicts the problem studied in this paper, where precise classification of even outlier classes in the target domain distribution is equally important.

**Figure 9:**
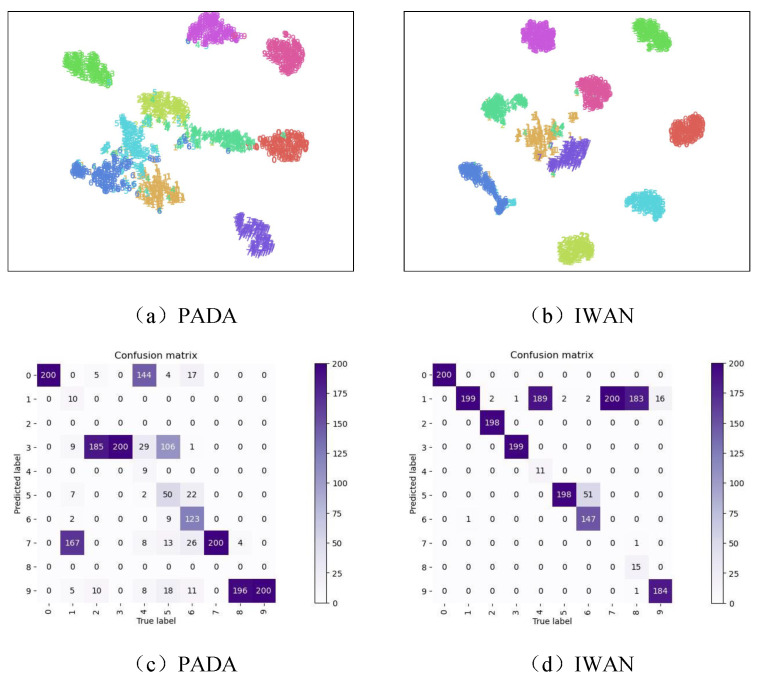
Feature distribution and confusion matrix of PADA and IWAN.

Figure 9 visually demonstrates the performance of M5 and M6 models using T-SNE technology and presents their confusion matrices. It can be observed that since classes 0, 3, 7, and 9 in the target domain are labeled during the training phase, their classification performance during the test phase is also quite good, as expected. However, the performance of the two models in classifying other classes is less than satisfactory. Through the T-SNE images, it can be seen that there are varying degrees of overlap and confusion among the features of these classes. Despite having a sufficient number of source domain samples for these classes during training, the poor classification performance seems to indicate that they were not adequately trained.

## 5. Conclusions

This paper focuses on addressing the challenge of rolling bearing diagnosis under different working conditions in real-world scenarios, starting from practical needs and deeply analyzing the shortage of data in real scenes, which reveals the limitations of current bearing fault diagnosis methods based on transfer learning. this paper focuses on exploring the scenario of label imbalance fault diagnosis, involving common challenges such as domain shift and label imbalance in bearing fault diagnosis, which collectively reflect the complexity of practical application scenarios. Faced with these challenges, an inter-class feature transfer fault diagnosis method is proposed in this paper, which aims to achieve efficient fault diagnosis performance across the entire label space by using a source domain dataset and a target domain dataset facing the label imbalance problem. Compared with a variety of methods on two datasets, ICFT both achieved the highest performance (84.55%, 96.23%). At the same time, the poor performance of IWAN and PADA in this paper (64.52%, 54% and 71.91%, 50.51%) reveals another partial transfer learning method that does not focus on outlier class information, which is different from the issue studied in this paper. The experimental results confirm the effectiveness and superiority of the proposed method, which provides a new effective solution for the field of rolling bearing intelligent fault diagnosis.

## Figures and Tables

**Table 1:** Fault classes and labels.

	**Class Label**	0	1	2	3	4	5	6	7	8	9
	**Fault Location**	0	IF	IF	IF	BF	BF	BF	OF	OF	OF
**Dataset I** ** Dataset II**	**Fault Size (mm)**	N/A	0.2	0.4	0.6	0.2	0.4	0.6	0.2	0.4	0.6

**Table 2:** Detailed information on each domain as the source and target domains.

	Domain	Load	Available Labels (as Source Domain)	Available Labels (as Target Domain)	Sample Size (as Source Domain)	Sample Size (as Target Domain)	Test Sample Size
Dataset I	A	0 N	0, 1, 2, 3, 4, 5, 6, 7, 8, 9	0, 3, 7, 9	200	200	200
	B	20 N	0, 1, 2, 3, 4, 5, 6, 7, 8, 9	0, 3, 7, 9	200	200	200
	C	40 N	0, 1, 2, 3, 4, 5, 6, 7, 8, 9	0, 3, 7, 9	200	200	200
	D	60 N	0, 1, 2, 3, 4, 5, 6, 7, 8, 9	0, 3, 7, 9	200	200	200
Dataset II	A	1 kN	0, 1, 2, 3, 4, 5, 6, 7, 8, 9	0, 3, 7, 9	200	200	200
	B	2 kN	0, 1, 2, 3, 4, 5, 6, 7, 8, 9	0, 3, 7, 9	200	200	200
	C	3 kN	0, 1, 2, 3, 4, 5, 6, 7, 8, 9	0, 3, 7, 9	200	200	200

**Table 3:** The method involved in the experiment.

Method	Description
M1	Only source domain data are used for training, and CE loss function is used in the training process
M2	The CE loss function is used to train the data of source domain and target domain
M3	CE + MMD
M4	DANN + Labeled target domain sample, CE loss
M5	IWAN + Labeled target domain sample, CE loss
M6	PADA + Labeled target domain sample, CE loss
M7	Minimum Class Confusion (MCC) [30] + Unlabeled target domain sample, CE loss
M8	MCC + Labeled target domain sample, CE loss
A1	Only AM-Softmax loss function is used
ICFT	The proposed complete method

**Table 4:** Fault diagnosis accuracy on Dataset I (%), Bold means best performance.

Task	M1	M2	M3	M4	M5	M6	M7	M8	A1	ICFT
A→B	58.80 ± 2.93	73.40 ± 1.94	74.03 ± 1.53	53.23 ± 5.81	54.88 ± 2.79	47.31 ± 1.03	49.44 ± 2.42	58.33 ± 1.24	73.22 ± 1.77	**77.09 ± 2.27**
A→C	50.73 ± 2.95	68.38 ± 0.34	68.6 ± 0.31	48.57 ± 4.95	49.32 ± 2.10	45.61 ± 1.76	41.39 ± 1.94	49.44 ± 1.11	68.22 ± 0.86	**69.85 ± 1.61**
A→D	51.48 ± 2.39	65.27 ± 0.68	67.45 ± 0.93	47.01 ± 6.13	47.73 ± 3.94	45.41 ± 2.08	58.06 ± 1.94	62.5 ± 1.39	65.2 ± 0.91	**67.77 ± 1.97**
B→A	54.56 ± 2.87	69.02 ± 0.42	69.61 ± 0.50	47.32 ± 4.00	49.06 ± 4.33	48.86 ± 1.35	58.06 ± 2.30	66.11 ± 1.11	70.44 ± 2.36	**70.53 ± 0.66**
B→C	95.29 ± 2.13	**99.88 ± 0.06**	99.83 ± 0.10	81.81 ± 2.56	84.53 ± 3.09	76.99 ± 2.22	97.22 ± 2.15	99.44 ± 1.11	99.60 ± 0.26	99.86 ± 0.09
B→D	88.25 ± 2.70	99.07 ± 0.22	98.72 ± 0.17	74.56 ± 3.76	78.03 ± 3.91	62.51 ± 6.41	91.11 ± 1.11	**99.44 ± 1.11**	97.83 ± 1.66	98.51 ± 0.54
C→A	44.7 ± 1.98	66.67 ± 0.56	65.52 ± 0.37	38.15 ± 2.52	41.27 ± 4.77	43.71 ± 1.54	48.61 ± 2.85	62.78 ± 1.36	65.18 ± 1.26	**67.13 ± 0.47**
C→B	93.76 ± 2.33	99.02 ± 0.17	99.44 ± 0.16	83.08 ± 2.53	80.68 ± 4.17	52.31 ± 2.69	92.78 ± 2.54	95.00 ± 1.67	99.2 ± 0.27	**99.71 ± 0.12**
C→D	96.66 ± 0.88	99.80 ± 0.09	99.84 ± 0.04	84.35 ± 2.39	85.22 ± 2.51	64.12 ± 3.21	98.05 ± 1.78	**100.00 ± 0.00**	99.75 ± 0.09	99.82 ± 0.06
D→A	50.23 ± 2.09	63.87 ± 0.69	66.12 ± 1.01	39.38 ± 3.56	42.31 ± 4.76	42.39 ± 0.85	47.78 ± 1.11	62.22 ± 1.36	63.59 ± 1.51	**66.85 ± 0.43**
D→B	84.88 ± 3.71	96.77 ± 0.38	98.21 ± 0.35	73.81 ± 2.78	75.49 ± 2.93	52.52 ± 3.05	84.17 ± 3.05	90.28 ± 3.11	96.05 ± 0.99	**98.74 ± 0.21**
D→C	95.6 ± 1.16	**98.78 ± 0.47**	97.49 ± 0.34	83.65 ± 2.05	85.73 ± 3.98	66.24 ± 5.33	97.22 ± 0.00	97.22 ± 0.00	97.8 ± 0.82	98.71 ± 0.49
Average	72.08	83.33	83.74	62.91	64.52	54.00	71.99	78.56	83.00	**84.55**

**Table 5:** Fault diagnosis accuracy on Dataset II (%), Bold means best performance.

Task	M1	M2	M3	M4	M5	M6	M7	M8	A1	ICFT
A→B	79.02 ± 1.23	87.10 ± 0.61	88.30 ± 1.30	87.47 ± 0.26	66.26 ± 4.18	48.74 ± 2.99	84.16 ± 1.78	**100.00 ± 0.00**	87.59 ± 0.30	94.14 ± 3.70
A→C	82.96 ± 1.05	86.96 ± 0.49	84.62 ± 0.59	87.19 ± 2.45	58.88 ± 4.34	48.62 ± 1.29	78.61 ± 1.27	85.28 ± 1.27	90.10 ± 2.22	**91.69 ± 3.16**
B→A	88.85 ± 0.12	98.69 ± 0.19	97.41 ± 0.45	98.71 ± 0.34	75.74 ± 4.09	47.07 ± 1.32	83.33 ± 0.00	**100.00 ± 0.00**	98.65 ± 0.18	98.89 ± 0.31
B→C	97.61 ± 0.21	97.05 ± 0.24	91.82 ± 1.10	96.66 ± 0.63	83.47 ± 4.39	54.53 ± 1.62	94.72 ± 1.50	93.05 ± 1.385	97.02 ± 0.37	**97.76 ± 0.82**
C→A	87.67 ± 0.10	97.03 ± 0.22	94.44 ± 1.03	96.30 ± 1.15	67.03 ± 8.32	43.32 ± 1.90	73.61 ± 1.39	90.56 ± 1.36	97.54 ± 0.18	**97.59 ± 0.40**
C→B	**97.77 ± 0.10**	97.65 ± 0.18	95.93 ± 0.56	97.69 ± 0.52	80.08 ± 3.16	60.76 ± 3.91	93.33 ± 1.36	99.72 ± 0.83	97.31 ± 0.34	97.31 ± 0.36
Average	88.98	94.08	92.09	94.00	71.91	50.51	84.63	94.77	94.70	**96.23**

## Data Availability

Data are unavailable due to privacy restrictions.
